# Retention of the Cluster Core Structure during Ligand Exchange Reactions of Carboxylato-Substituted Metal Oxo Clusters

**DOI:** 10.1002/ejic.201403209

**Published:** 2015-03-18

**Authors:** Johannes Kreutzer, Michael Puchberger, Christine Artner, Ulrich Schubert

**Affiliations:** [a] Institute of Materials Chemistry, Vienna University of TechnologyGetreidemarkt 9, 1060 Vienna, Austria

**Keywords:** Cluster compounds, Cage compounds, Ligand exchange, Zirconium, Carboxylate ligands

## Abstract

The exchange of the carboxylato ligands of Zr_4_O_2_(methacrylato)_12_ in reactions with carboxylic acids proceeds with retention of the composition and structure of the cluster core. This was concluded from exchange/re-exchange experiments and from comparison of the IR bands of the cluster core of the original and ligand-exchanged clusters. The IR bands were assigned on the basis of DFT calculations. Scrambling reactions between two Zr_4_O_2_(OOCR)_12_ clusters with different carboxylato ligands are a new way to prepare mixed-ligand oxo clusters.

## Introduction

Transition metal oxo clusters are used as structurally well-defined nanosized building blocks for the synthesis of organic–inorganic hybrid materials.[1,2] Carboxylato-substituted oxo clusters are readily prepared through the reactions of metal alkoxides and carboxylic acids.[3] The carboxylic acids not only provide carboxylato ligands but also act as an in situ water source through esterification with the eliminated alcohol.

Postsynthesis ligand exchange reactions are important for the use of such clusters in materials syntheses and are model reactions for nanoparticles. For example, the exchange of monocarboxylato ligands with bridging dicarboxylato ligands has been used for the synthesis of metal–organic framework (MOF) structures with clusters as preformed building blocks.[4,5] When clusters (with unsaturated carboxylato ligands) are polymerized with organic co-monomers, cluster-crosslinked polymers are obtained. The modification of the ligand shell of the employed clusters by postsynthesis ligand exchange reactions allows the modification of the properties of the derived hybrid materials.[3,6]

This modification of the ligand shell is commonly achieved by reactions between the clusters and carboxylic acids, and both partial or complete exchange of the original carboxylato ligands has been demonstrated.[6–8] In this paper, we will show that scrambling reactions of clusters with the same cluster core but different carboxylato ligands are another possible method for the synthesis of mixed-ligand oxo clusters.

A question that has to be checked in every case is whether the exchange of coordinatively bonded ligands occurs with retention of the cluster core composition and structure. Although several successful exchange reactions have been reported for metal oxo clusters,[4–9] the rearrangement or degradation of the cluster core has also been observed.[10] The risk that the cluster rearranges or degrades upon ligand exchange is minimized if the leaving and entering ligands have the same charge and occupy the same number of coordination sites.[11]

**Figure 1:**
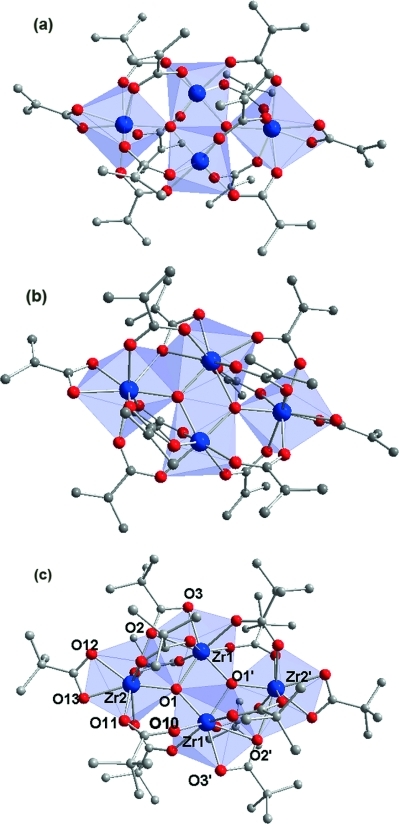
The two crystallographically determined molecular structures of Zr4 [(a) Zr4(sym) and (b) Zr4(asym)] and the molecular structure of Zr4Piv (c). Selected bond lengths [pm] and angles [°] for Zr4Piv: Zr1–O1 208.8(8), Zr1–O1′ 207.6(2), Zr2–O1 201.9(2), Zr1–O2 240.4(6), Zr1–O3 221.3(6), Zr2–O2 248.1(1), Zr2–O12 220.1(6), Zr2–O13 227.1(9), Zr2–O11 218.2(1), Zr1′–O10 218.1(1). Zr1–O1–Zr2 117.5(9), Zr1′–O1–Zr2 134.5(4), Zr1–O1–Zr1′ 106.2(2), Zr1–O2–Zr2 91.9(7), O12–Zr2–O13 57.15, O2–Zr1–O3 56.5(7), O1–Zr2–O12 147.8(6), O1–Zr2–O13 154.5(6), O1–Zr1–O2 75.4(3), O1–Zr2–O2 74.8(7), O1–Zr1–O3 131.3(7), O1–Zr2–O11 92.7(6), O1–Zr1′–O10 81.8(9).

The confirmation of the integrity of the cluster core in metal oxo clusters is not trivial in many cases, owing to the lack of suitable and easy to apply spectroscopic methods. In this article, we compare several possible solutions to this issue. The previously reported cluster Zr_4_O_2_(OMc)_12_ (Zr4, OMc = methacrylato; Figure1, a and b) was used for proof of concept.[12,13]

## Results and Discussion

The exchange reactions of coordinatively bonded ligands (L, L′), such as carboxylato ligands, are equilibria [Equation (1) for anionic ligands L and L′] and, therefore, the composition of the system can be shifted to one side or the other by changing the concentrations of H–L or H–L′. The required excess of H–L or H–L′ depends on the magnitude of the equilibrium constant. If the cluster core (M*_x_*O*_y_*) is retained during the exchange reaction [left to right in Equation (1)], then re-exchange of the ligands L′ of isolated M*_x_*O*_y_*L_*z*–*n*_L′*_n_* [right to left in Equation (1)] should result in the original cluster M*_x_*O*_y_*L*_z_*.

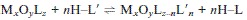
(1)

Such an exchange/re-exchange experiment (see also ref.[7]) was performed for Zr4 and pivalic acid. A CH_2_Cl_2_ solution of Zr4 was treated with 350 equiv. of pivalic acid. The large excess was used to guarantee complete exchange of the ligands. This resulted in the formation of Zr_4_O_2_(OPiv)_12_ (Zr4Piv, OPiv = pivalato), the molecular structure of which is shown in Figure1c. The cluster core of Zr4Piv is the same as that of Zr4, and the relevant bond lengths and angles are in close agreement with each other (Table1). Two molecular structures of Zr4 have been crystallographically determined; they have the same cluster core but slightly different ligand arrangements. The isomers Zr4(sym) and Zr4(asym) give the same NMR spectra in solution, that is, the different coordination of one OMc ligand only occurs in the crystal structure. In centrosymmetric Zr4(sym),[13] ten of the OMc ligands are bridging and two are chelating (Figure1, a). In Zr4(asym).[12] one of the OMc ligands is chelating–bridging (η^2^,μ_2_) instead. In contrast, two OMc ligands in centrosymmetric Zr4Piv are chelating–bridging (O2 bridging Zr1 and Zr2, O2 and O3 chelating Zr1).

**Table 1:** Bond lengths [pm] and bond angles [°] of the cluster cores of Zr4 and Zr4Piv (the atom numbering refers to the structure of Zr4Piv).

Bond	Zr4_sym_	Zr4_asym_	Zr4Piv	Bonds	Zr4	Zr4_asym_	Zr4Piv
Zr1–O1	205.2(5)	205.6(4)	208.8(9)	Zr1′–O1–Zr2	122.6(3)	123.6(3)	117.5 (9)
Zr1′–O1	211.2(3)	217.3(6)	207.6(2)	Zr1–O1–Zr2	132.1(9)	122.9(1)	134.5(4)
Zr2–O1	206.5(2)	203.0(1)	201.9(1)	Zr1–O1–Zr1′	104.5(6)	104.8(4)	106.2(2)

**Figure 2:**
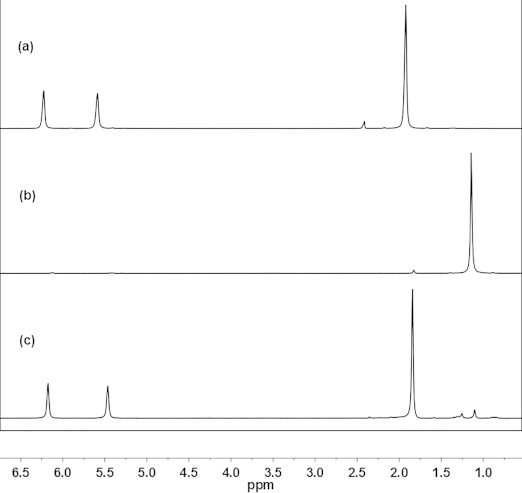
Room-temperature ^1^H NMR spectra (CDCl_3_) of (a) original Zr4, (b) Zr4Piv (after ligand exchange), and (c) Zr4 after re-exchange. The signal at *δ* = 2.3 ppm is due to residual toluene from washing.

The room-temperature ^1^H NMR spectrum (Figure2, a) of Zr4 shows only three singlets owing to ligand dynamics, namely, a singlet at *δ* = 1.92 ppm for the methyl group of the methacrylato ligands and two signals at *δ* = 6.22 and 5.58 ppm for the methylene group. Analogous behavior was observed for Zr4Piv: a singlet at *δ* = 1.16 ppm (Figure2, b) indicates that the pivalato ligands in Zr4Piv also undergo dynamic site exchange.

**Figure 3:**
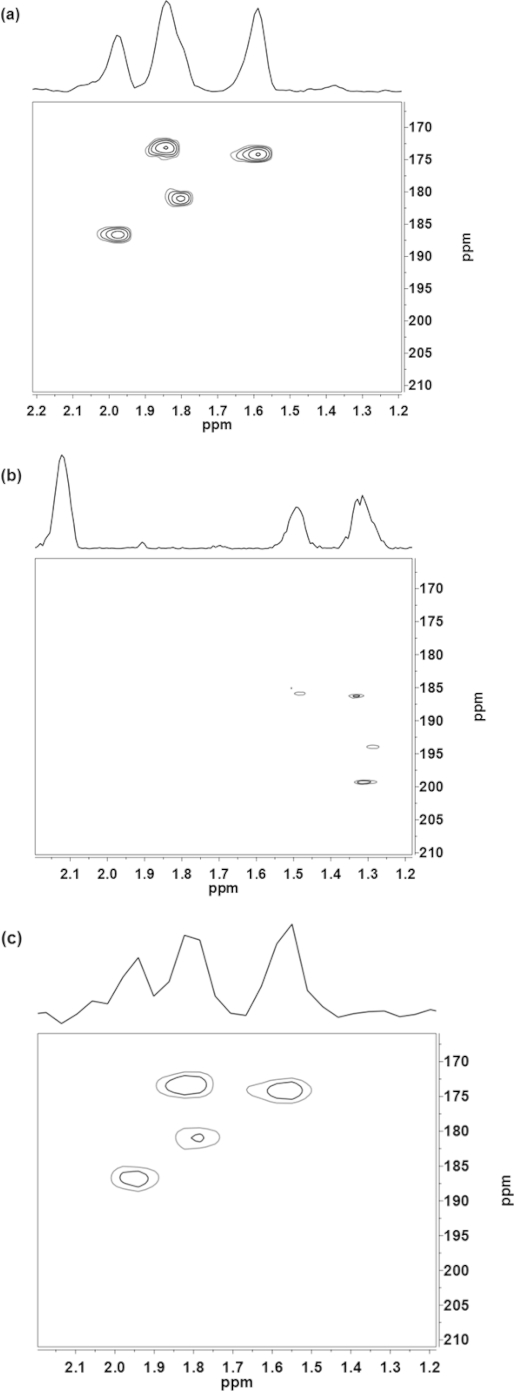
^1^H–^13^C HMBC spectra ([D_8_]toluene, –80 °C) of (a) Zr4, (b) Zr4Piv (crystals obtained from the exchange experiment), and (c) Zr4 after re-exchange.

The ^1^H–^13^C HMBC spectrum of Zr4 at –80 °C (see Figure3, a) shows four sets of chemically inequivalent signals in agreement with the *C*_2*h*_ molecular symmetry of Zr4 (sym) as has been discussed previously.[14] The three highfield peaks (*δ* =173.2, 174.2, and 181.1 ppm) correspond to the bridging OMc ligands of Zr4, and the fourth peak at *δ* = 186.7 ppm is attributed to the chelating ligand. The same splitting into four sets of peaks is found for the Zr4Piv cluster (Figure3, b). Here, the bridging pivalato ligands give cross-peaks at *δ* = 185.89, 186.28, and 193.96 ppm, and the chelating pivalato ligand gives a cross-peak at *δ* = 199.3 ppm (see Figure3, b).

To prove the reversibility of the ligand exchange, the pivalato ligands of isolated Zr4Piv were re-exchanged with methacrylato ligands by multiple treatment of Zr4Piv with a large excess of methacrylic acid. The room-temperature ^1^H NMR spectrum of the cluster obtained after re-exchange (Figure2, c) was identical to that of the original Zr4 cluster. The same is true for the HMBC spectrum of Zr4 after the re-exchange of all of the pivalato ligands with methacrylato ligands (Figure3, c).

The exchange reactions of carboxylato-substituted oxo clusters and carboxylic acids [Equation (1)] were proposed to proceed through change of the coordination of the original carboxylato ligand (L) from η_2_ to η_1_, the addition of H–L′ to the vacated coordination site, proton transfer between the entering (L′) and leaving (L) carboxylato groups, elimination of HL, and change of the coordination of L′ from η_1_ to η_2_.[6] According to the experiments described above, this sequence of events for Zr4 is apparently possible without any changes to the structure of the cluster core.

As shown previously for another Zr cluster type, stepwise ligand exchange is possible; thus, clusters with two types of carboxylato ligand can be prepared.[6] According to the results reported above, the mixed-ligand clusters Zr_4_O_2_(OMc)_12–*x*_(OPiv)*_x_* should have the same structure as that of Zr4 and Zr4Piv. We tested another possibility for ligand exchange, that is, ligand scrambling between two different clusters, each comprising only one ligand type [Equation (2), with L and L′ = carboxylato groups]. However, the mechanism must be different to that of Equation (1) because no protic compounds are involved. A plausible mechanism would be the intermediate formation of carboxylato bridges between the two cluster cores; the question is whether the core structure would be retained in such a process.



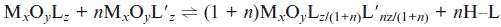
(2)

**Figure 4:**
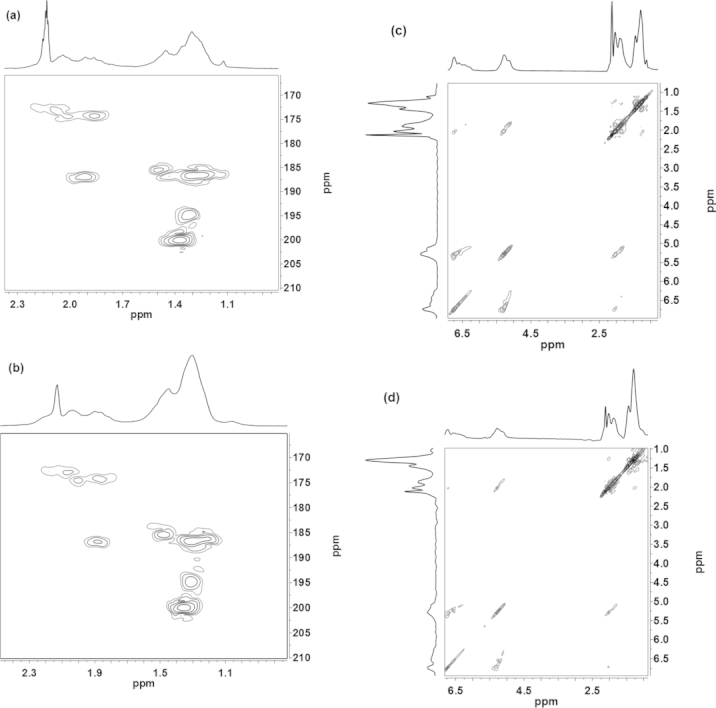
^1^H–^13^C HMBC spectra of Zr_4_O_2_(OMc)_6_(OPiv)_6_ obtained by (a) ligand exchange of Zr4 and (b) by scrambling reaction between Zr4 and Zr4Piv in toluene; NOESY spectra of Zr_4_O_2_(OMc)_6_(OPiv)_6_ obtained by (c) ligand exchange and (d) by scrambling between Zr4 and Zr4Piv in toluene. All spectra were recorded at –80 °C with samples in [D_8_]toluene.

To investigate this possibility, Zr4 and Zr4Piv in a 1:1 molar ratio were dissolved in [D_8_]toluene, and the ^1^H–^1^H NOESY and ^1^H–^13^C HMBC spectra of the resulting compounds were recorded (Figure4). For comparison, Zr_4_O_2_(OMc)_6_(OPiv)_6_ was prepared by exchanging 50 % of the OMc ligands of Zr4 with pivalato ligands by reaction with pivalic acid, as described above. The ^1^H–^13^C HMBC spectrum of Zr_4_O_2_(OMc)_6_(OPiv)_6_ is a superposition of those of Zr4 and Zr4Piv. It shows the same peak pattern independent of the preparation protocol, that is, by ligand exchange between Zr4 and pivalic acid (Figure4, a) or by scrambling between Zr4 and Zr4Piv (Figure4, b). The ^1^H–^1^H NOESY spectrum of Zr_4_O_2_(OMc)_6_(OPiv)_6_ showed NOE signals between the methyl groups of the methacrylato ligands and the methyl groups of the pivalato ligands (Figure4, c). This proves that both ligands are bonded to the same cluster. The NOESY spectrum of the scrambling experiment (Figure4, d) was identical, that is, the same cluster was formed. From the experiments described above, it can be concluded that the mixed-ligand cluster Zr_4_O_2_(OMc)_6_(OPiv)_6_ must have the same structure as that of Zr4 and Zr4Piv independent of the preparation method.

Infrared spectroscopy is undoubtedly a very convenient characterization method, but unfortunately the characteristic bands of the cluster core [in the mind-IR (MIR) and far-IR (FIR) regions] are difficult to identify, and the bands of the ligands provide no relevant information. Therefore, we performed DFT calculations on Zr4 with the crystallographically determined structure of Zr4 (sym) as the input.[12] The thus identified bands of the cluster core were then used for comparison with those of the cluster obtained after ligand exchange, Zr4Piv.

**Figure 5:**
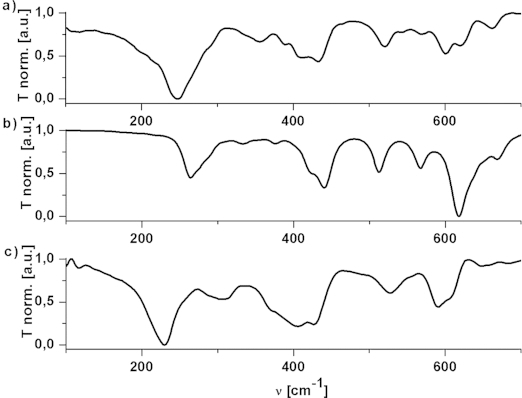
ATR-IR and calculated IR spectra in the MIR/FIR region of (a) Zr_4_ (sym) experimental, (b) Zr_4_ calculated, and (c) Zr_4_Piv experimental.

The agreement between the calculated and experimental spectrum of Zr4 is sufficiently good in terms of band position and band shape. The unscaled calculated frequencies are shifted slightly by ca. 5 % to higher wavenumbers (Table2 and Figure5). The IR bands of the methacrylato cluster Zr4 were assigned on the basis of the calculations and compared with those of the pivalato cluster Zr4Piv. The carboxylato stretching vibrations give strong bands in the range 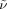 = 1600–1350 cm^–1^. Two additional strong bands at 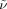 = 1100 and 1000 cm^–1^ were observed for Zr4Piv. The bands that support the intactness of the core are the asymmetric stretching vibration of the μO bridges of Zr4 at 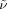 = 520 cm^–1^ (512 cm^–1^ calculated), which is only slightly shifted to 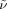 = 524 cm^–1^ for Zr4Piv. The bands of the symmetric and asymmetric stretching of the Zr-μO core of Zr4 were observed at 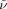 = 247 (calculated 263 cm^–1^) and 292 cm^–1^ (276 cm^–1^ calculated), respectively, and were shifted to 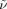 = 228 and 257 cm^–1^ for Zr4Piv. The agreement of the most prominent IR bands of Zr4 and Zr4Piv, in particular the good agreement of the core vibrations, showed that the core was also preserved during the exchange reactions.

**Table 2:** Experimental (ATR-IR) and calculated IR bands; b = bridging, c = chelating carboxylato ligands; ω wagging vibration, τ twisting vibration, δ deformation vibration. R = CH_2_ for Zr4 and Me_2_ for Zr4Piv.

Zr4	Calcd.	Zr4Piv	Assignment	Zr4	Calcd.	Zr4Piv	Assignment
2978	3149	2960	ν_as_(Me)	1009	1027		ω(Me)
2926	3062	2928	ν_s_(Me)	940	979		ω(CH_2_)
1646	1732		ν_as_(CMeCH_2_)	663	670		τ(CH_2_)
1582	1683	1579	ν_as_(COO)^b^	601/621	619	591/606	ν_s_(COO)^b^
1559	1662		ν_as_(COO)^b^	569	567		ν_as_(COO)^b^
1495	1578		ν_as_(COO)^c^	520	512	523	δ_s_(μO–Zr)
1459	1515		ν_s_(COO)^c^	433	438	425	ν(CMeR)
1419	1498	1485	ν_s_(COO)^b^	405	420	402	ν(CMeR)
1371	1473	1361	ν_s_(COO)^b^	292	276	257	δ_as_(μO–Zr)
1239	1280	1228	ν_s_(CMeR)	247	263	228	δ_s_(μO–Zr)

## Conclusions

We have compared several possibilities to check the integrity of the cluster core during ligand exchange reactions. The (complete or partial) exchange of the carboxylato ligands of Zr_4_O_2_(OMc)_12_ (Zr4) is a reversible process. The exchange/re-exchange experiment showed that the cluster core stays intact even when Zr4 is treated with a large excess of carboxylic acid. The integrity of the cores of Zr_4_O_2_(carboxylato)_12_ clusters was also confirmed by FIR spectroscopy after assignment of the core vibrations on the basis of DFT calculations.

Scrambling reactions between Zr_4_O_2_(carboxylato)_12_ clusters with different carboxylato ligands provide an alternative method to prepare mixed-ligand clusters. The reaction between Zr4 and Zr4Piv resulted in the same mixed-ligand cluster as that from the partial exchange of the methacrylato ligands of Zr4 with pivalato ligands. The advantage of scrambling reactions is the possibility to predetermine the composition of the ligand sphere through the mixing ratio of the two clusters. This makes it possible to adjust the ratio of functional and nonfunctional ligands on the cluster surface. Furthermore, no purification steps are necessary after scrambling reactions as no free carboxylic acid is involved. The straightforward adjustment of the ligand ratio is a big advantage compared to ligand exchange reactions between a cluster and carboxylic acids, for which the number of exchanged ligands depends on the equilibrium constant of the exchange reaction, and this is not known for most carboxylato cluster combinations.

## Experimental Section

**General Methods:** All operations were performed under a nitrogen atmosphere by standard Schlenk techniques. Hexane and toluene were dried with Na/benzophenone before use. Dichloromethane was distilled from CaH_2_. Methacrylic acid (99 %) and pivalic acid were obtained from Sigma–Aldrich and freshly distilled from P_2_O_5_ before use. Zr_4_O_2_(OMc)_12_ (Zr4) was prepared as previously reported.[12] The crystalline product was dried under vacuum, dissolved in a small amount of CH_2_Cl_2_ until a clear solution was obtained, and precipitated from hexane. This procedure was repeated three times to remove free acid.

**Computational Details:** The DFT calculations were performed with the Gaussian09 program.[15] The PBE0 functional[16] in combination with the scalar relativistic Stuttgart–Dresden effective core potential (SDD)[17] for Zr and the polarized triple-ζ basis set (TZVP) from Ahlrich[18] for light elements (H, C, O) was employed in all calculations. Optimization without symmetry constraints started from the experimental crystal structure of Zr4(sym).[13] The relaxed geometry shows mean average errors of 0.01 Å for the bond lengths and 0.53° for the bond angles. The bond lengths are slightly elongated, and the angles appear narrower in the calculated structure. The optimized geometry was confirmed as a minimum structure by the absence of imaginary frequencies. The frequencies of the IR calculations were not scaled. The spectra were simulated with a Lorentzian broadening with a half-width of 10 cm^–1^.

**Characterization Techniques:** Solution ^1^H NMR spectra were recorded with a Bruker AVANCE 250 spectrometer (250.13 MHz for ^1^H, 62.86 MHz for ^13^C). The 2D spectra were recorded with a Bruker AVANCE 300 spectrometer (^1^H at 300.13 MHz, ^13^C at 75.13 MHz) equipped with a 5 mm inverse probehead with a *z* gradient unit, and a Bruker standard pulse sequence was used. Gas-tight Young tubes were used for all experiments. CDCl_3_ and [D_8_]toluene were purchased from Euriso-Top and degassed by freeze–pump–thaw cycles.

Solid-state attenuated total reflectance IR (ATR-IR) spectra were recorded with a Perkin–Elmer Spectrum 400 FTIR spectrometer equipped with a KBr window for MIR and a polyethylene window for FIR; 128 scans were averaged for MIR measurements, and 256 scans were averaged for FIR measurements. The spectra were processed with the Perkin–Elmer Spectrum software and normalized with the implemented normalization routine.

**X-ray Structure Analysis:** All measurements were performed at 100 K with Mo-*K_α_* (*λ* = 71.073 pm) radiation. The data were collected with a Bruker AXS SMART APEX II four-circle diffractometer with κ-geometry through *φ* and *ω* scans and different frame widths. The data were corrected for polarization and Lorentz effects, and an empirical absorption correction (SADABS) was employed. The cell dimensions were refined with all unique reflections. The SAINT PLUS software (Bruker Analytical X-ray Instruments) was used to integrate the frames. Details of the X-ray investigation are given in Table3.

**Table 3:** Crystal data and refinement details for Zr4Piv.

Empirical formula	C_60_H_106_O_26_Zr_4_
*M*_r_	1610.34
Crystal system	monoclinic
Space group	*P*2_1_/*c*
*a* /pm	1871.6(6)
*b* /pm	2080.3(9)
*c* /pm	1930.9(4)
*β* /°	91.278(3)
*V* /pm^3^ × 10^6^	7516.8
*Z*	4
*D*_calcd._ /Mg m^–3^	1.423
*μ* /mm^–1^	0.611
Crystal size /mm	0.37 × 0.28 × 0.26
Measured reflections	133890
Observed reflections [*I* > 2σ(*I*)]	8479
*θ*_max_ /°	22.83
*R* [*F*^2^ > 2σ(*F*)], *wR* (*F*^2^), *S*	0.1061, 0.3218, 1.183
Reflections/parameters	9992/860
Weighting scheme[a]	*w* = 1/[*σ*^2^(*F*_o_^2^) + (0.0934*P*)^2^ + 362.3243*P*]
*ρ*_max_, *ρ*_min_ /e × 10^–6^ pm^–3^	3.131, –1.880

[a] *P* = (*F*_o_^2^ + 2*F*_c_^2^)/3.

The structures were solved by the Patterson method (SHELXS-97).[19] Refinement was performed by the full-matrix least-squares method based on *F*^2^ (SHELXL-97) with anisotropic thermal parameters for all non-hydrogen atoms. Hydrogen atoms were inserted in calculated positions and refined as riding with the corresponding atom. The crystal diffracted only weakly. Relatively high electron density remained (3.13 and 2.65 e × 10^–6^ pm^–3^) between the Zr and O atoms. Furthermore, some of the *t*Bu groups of the pivalato ligands showed positional disorder.

CCDC-1038630 contains the supplementary crystallographic data for this paper. These data can be obtained free of charge from The Cambridge Crystallographic Data Centre via www.ccdc.cam.ac.uk/data_request/cif.

**Ligand Exchange:** Pivalic acid (5.57 mL, 49.35 mmol) was added dropwise to a solution of Zr4 (200 mg, 0.141 mmol) in CH_2_Cl_2_ (10 mL), and the mixture was stirred for 30 min. After the removal of the solvent in high vacuum (10^–6^ mbar), the residue was redissolved in CH_2_Cl_2_ (0.5 mL) and precipitated from acetonitrile (50 mL). The solution was centrifuged, and the solvent was decanted. The obtained solid was dried under vacuum.

Crystals of Zr4Piv were obtained by recrystallization but had poor quality; therefore, the structure could not be refined sufficiently. More-suitable crystals were obtained directly from Zr(O*i*Pr)_4_ and pivalic acid.

Methacrylic acid (2.63 mL, 31.04 mmol) was added dropwise to a solution of Zr4Piv (100 mg, 0.062 mmol) in CH_2_Cl_2_ (5 mL). The solution was stirred for 30 min, and then the solvent was removed under vacuum. The obtained solid was dissolved in dichloromethane (0.5 mL) and precipitated from hexane (50 mL). This procedure was applied three times.
